# Fatal hemolytic uremic syndrome associated with day care surgery and anaesthesia: a case report

**DOI:** 10.1186/1756-0500-6-242

**Published:** 2013-06-26

**Authors:** Anna Myrnäs, Markus Castegren

**Affiliations:** 1 Department of Anaesthesia, Intensive Care Medicine, Sörmland County Hospital/Mälarsjukhuset, Eskilstuna, Sweden; 2 Centre for Clinical Research Sörmland, Uppsala University, Kungsgatan 41, Eskilstuna, Uppsala, SE-63188, Sweden; 3 Department of Medical Sciences, Infectious Diseases, Uppsala University, Uppsala, Sweden

**Keywords:** Thrombotic microangiopathy, Thrombotic thrombocytopenic purpura, Plasmapheresis, Day care surgery, General anaesthesia

## Abstract

**Background:**

Thrombotic angiopathies, i.e. haemolytic uremic syndrome and thrombotic thrombocytopenic purpura, are thought to occur in patients with a combination of risk factors (e.g., an infection with shiga-toxin-producing Escherichia coli (E. coli) or low activity of the metalloproteinase Adamts-13) and a pathophysiological trigger (e.g., anti-endothelial antibodies, cytokines or activation of chemokine receptor 4). To our knowledge, this is the first report describing an association between haemolytic uremic syndrome and routine surgery and anaesthesia.

**Case presentation:**

We present a case in which a 67-year-old Caucasian female developed fatal haemolytic uremic syndrome in the immediate postoperative period of uncomplicated day care surgery. The patient had suffered gastrointestinal symptoms followed by confusion approximately two weeks before surgery, but had been without any symptoms in the week before surgery. Haemolytic uremic syndrome with cerebral symptoms ranging from initial anxiety to subsequent seizures and coma developed within a few hours after the end of surgery. In addition, acute kidney failure and severe thrombocytopenia occurred about the same time. During intensive care, the patient was found to be positive for enterohaemorrhagic E. coli (EHEC) in faeces.

**Conclusion:**

Anaesthesiologists should be notified that haemolytic uremic syndrome is an uncommon differential diagnosis in patients with postoperative seizures and coma. Patients with a recent enterohemmoragic E.Coli infection should be followed postoperatively for signs of haemolytic uremic syndrome.

## Background

Haemolytic uremic syndrome (HUS) is characterised by three prominent attributes: haemolytic anaemia, acute kidney failure and thrombocytopenia [1]. The most common cause of classical HUS (STEC-HUS) is infection with shiga-toxin-producing Escherichia coli (E. coli) 0157:H7. Typically, the incubation period is 3–5 days and approximately 5-15% of infected patients develop HUS. Children are more often affected than adults. Neurological involvement is uncommon [2]. During the German major outbreak of HUS in 2011, patients were infected with E. coli O104:H4. During the outbreak, over 20% of the infected patients developed HUS and 88% of the cases with HUS occurred in adults. Particularly noteworthy was that 68% of the patients were women [3]. Furthermore, neurological involvement was common, whereas the incidence of bloody diarrhoea was infrequent [4]. Neurological symptoms occurred at the same time as there was an increase of lactate dehydrogenase and creatinine and the nadir of the platelet count occurred before the onset of neurological symptoms [5]. The patients needed mechanical ventilation for an average of five days and started to be seizure free after an average of three days [5].

In patients in whom no infection with shiga-toxin-producing E. coli is found, i.e. atypical HUS (aHUS), mutations in genes encoding proteins of the alternative pathway of the complement system have been identified [6]. A third condition very similar in presentation and pathophysiology, thrombotic thrombocytopenic purpura (TTP), is associated with either a low activity of the metalloproteinase Adamts-13 or antibodies to the protein [7]. All three conditions are sometimes referred to as thrombotic microangiopathies (TMAs) [7].

In contrast to aHUS, which has a known risk of recurrence following renal transplantation [8], STEC-HUS is regarded as a unique event not associated with recurrence of the symptoms [9]. In the reports after the 2011 outbreak of STEC-HUS no comments about patients with recurrent presentations were noted [5,10].

Drug-associated HUS is a commonly acknowledged condition in which the proposed mechanisms are direct endothelial effects or immune-mediated effects associated with deficient Adamts-13 activity [11]. The most commonly implicated drugs are quinine, mitomycin-C, tacrolimus and cyclosporine, as well as platelet-acting drugs, such as clopidogrel and ticlopidine [12]. Other than the most common drugs associated with HUS, reports of a suspected association can be found between the development of HUS and treatment with antibiotics, i.e. penicillin and piperacillin, non-steroidal anti-inflammatory drugs, H_2_-receptor antagonists and simvastatin [11].

Adamts-13 protein deficiency is not enough for the development of TTP; rather, a pathophysiological trigger is required for the precipitation of the condition [13]. Anti-endothelial antibodies or cytokines have been proposed as such triggers [13]. An interplay between a predisposing condition and a pathophysiological trigger is most likely also required in STEC-HUS and aHUS, where activation of chemokine receptor 4 (CXCR4/stromal cell-derived factor-1(SDF-1) pathway) has been proposed as a trigger mechanism [14]. Ubiquitin, a natural CXCR4 agonist [15], is released systemically following trauma and inflammation [16].

Evidenced-based treatments for TMAs are lacking; however, plasma exchange therapy has been used with varying success [17]. Complement inhibitor treatment with the monoclonal antibody against the compliment protein C5, eculizumab, has been used successfully in recent trials in aHUS [6].

To our knowledge, there are no publications or case reports describing an association between STEC-HUS with uncomplicated anaesthesia and routine orthopaedic surgery.

## Case presentation

A 67-year-old Caucasian female with glipizide- and metformin-treated diabetes mellitus and enalapril-treated arterial hypertension presented for open reposition and Zuggurtung fixation of a fracture of the olecranon. The elbow was fractured in a bicycle accident and had been conservatively treated for 14 days prior to surgery. The patient had suffered from diarrhoea and signs of confusion in the period between the trauma and surgery, but had been free of gastrointestinal or neurological symptoms during the week before surgery. The patient had not sought professional medical advice for these symptoms. When the patient presented for surgery, she was lucid, without any gastrointestinal symptoms and otherwise physically well. The preoperative laboratory work-up tests were without pathological signs (Table 1). At the operating ward, 1 g paracetamol, 100 mg diclofenac, 10 mg oxycodone and 25 mg meclizine were administered orally as premedication. The anaesthesia was induced with propofol and fentanyl. The airway was secured by orotracheal intubation after which the anaesthesia was maintained with sevoflurane. The time of surgery was 31 min and the total anaesthesia duration was 70 min. After uncomplicated surgery and anaesthesia, the patient was transferred to the postoperative unit from where the patient went home after 3 h accompanied by her next of kin. During the first postoperative evening and night, the patient’s next of kin observed that the patient had problems with articulating words followed by increasing anxiety and confusion. At the next morning, the patient only spoke unrecognizable words, and by the afternoon, she could not walk. Approximately 24 h after leaving the hospital, the patient presented at the emergency ward. Respiration and circulation were normal and the patient was afebrile. A computed tomography (CT) scan of the brain was performed as well as an analysis of the cerebrospinal fluid, with both indicating no pathological signs. A neurological consult ordered an electroencephalogram (EEG) and a magnetic resonance imaging (MRI) of the brain to be performed as soon as possible at a secondary hospital to which the patient was referred. At arrival to the secondary hospital on the second postoperative day, the patient did not respond verbally, could not open her eyes spontaneously and the best motor response was withdrawal of the limbs on pain stimulation (Glasgow Coma Scale, GCS 9). It was noted that plasma creatinine was elevated to 313 μmol × L^-1^. Except for an elevated C-reactive protein (CRP) at 76 mg × L^-1^, all blood analyses were in the normal range, including haemoglobin, leucocyte and platelet count. On the night between the second and third postoperative day, the patient suffered a generalised tonic-clonic seizure. The patient was transferred to the intensive care unit, where she, was orotracheally intubated and mechanically ventilated. A second CT scan of the brain showed no bleeding or ischaemic signs. The patient was respiratory and circulatory stable and did not require an increased oxygen fraction or vasoactive drugs. The patient was anuric, was not icteric and did not have any pathological signs on the skin. Blood cultures were drawn and an antibiotic was given based on a body temperature of 38.9°C and tachycardia. The patient was started at 4 g piperacillin-tazobactam 3 times per day intravenously (i.v.). The cultures did not show any bacterial growth. Because the patient had elevated serum potassium of 5.8 mmol × L^-1^ and hyponatraemia of 131 mmol × L^-1^, blood was analysed for serum cortisol on the suspicion of Addison’s disease. Accordingly, the patient was given 100 mg hydrocortisone i.v. Cortisol in serum was found to be 796 nmol × L^-1^ and thus the suspicion of Addison’s disease was refuted. The patient was now anaemic with a haemoglobin of 86 g × L^-1^ and thrombocytopenic with a platelet count of 31 × 10^9^ × L^-1^. Lactate dehydrogenase was elevated to 21.5μkat × L^-1^ and haptoglobin low at 0.07 g × L^-1^, which indicate haemolysis. The blood film showed schistocytosis., On the third postoperative day, plasmapheresis was started on the indication of HUS/TTP. One plasma volume was replaced daily for four days and continuous veno-venous haemodiafiltration was performed between plasmapheresis treatments. The patient’s haemoglobin and platelet count improved during treatment (Table 1). The same positive development was seen for creatinine. Neurological status improved slowly and on the seventh postoperative day, GCS was 6 with withdrawal of the limbs on pain stimulation. Polymerase chain reaction (PCR) revealed enterohaemorrhagic E. coli (EHEC) in faeces. The serotype was non-O157, produced verotoxin type 2 and was eae-gene negative. Blood analysis of Adamts-13 protein activity showed normal levels and antibodies against the protein were not observed. Signs of multiple small ischaemic fronto-temporal cortical lesions were noted on an MRI scan, as well as lesions in the circulus Willisi and the basilar artery with narrowing and more distal dilatations of the vessels. Analyses of anti-neutrophil antibodies and anti-neutrophil cytoplasmic antibodies were negative. The neurological consult assessed the clinical picture together with the MRI findings as thrombotic microangiopathy. The patient continued to show slow improvement in neurological status with spontaneous eye opening and the ability to move all limbs, although with substantial weakness. The patient could make eye contact on instructions on the 20^th^ postoperative day but was still anuric and in need of intermittent haemodialysis and mechanical ventilatory support. On the 21^st^ postoperative day, the patient suffered a generalised tonic-clonic seizure followed by deep coma (GCS 3). EEG showed generalised deeply suppressed activity. A joint decision was made with the next of kin to withdraw all treatment. The patient died 6 h later.

**Table 1:** Development of the glasgow coma scale and laboratory parameters

**Days after surgery**	**Pre**-**operative analyses**	**1**	**2**	**4**	**6**	**13**
Glasgow coma scale	15	11	3		6	
Haemoglobin (g × L^-1^)	121	111	86	101	102	104
Platelet count (10^9^ × L^-1^)		112	31	84	113	389
Leukocyte count (10^9^ × L^-1^)		8.6	9.3	11	12.1	28.5
C-reactive protein (μmol × L^-1^)		76	134	59	9	30
Creatinine (μmol × L^-1^)	53	207	313	209	128	
Prothrombin time (INR)		1.1	1.1	1.1	1.1	1.1
Bilirubin total (μmol × L^-1^)			24		7	
Lactate dehydrogenase (μkat × L^-1^)			21.5		4	

## Discussion

The present case of a patient with gastrointestinal symptoms before the onset of HUS and positive PCR of EHEC in faeces is a classic case of HUS. The features of the E. coli being non-serotype O157, verotoxin 2-producing and eae-gene negative, together with the neurological symptoms in an adult female, are uncharacteristic of classical STEC-HUS but similar to the German outbreak of E. coli O104:H4 in 2011 [4]. In this case, the patient had suffered gastrointestinal symptoms followed with transient confusion and recovered from all symptoms over a week prior to surgery. However, in the immediate postoperative period, the cerebral symptoms recurred, only now with rapid deterioration from confusion and agitation to fulminant epileptic seizures and deep coma. The progression from a patient without symptoms before surgery and anaesthesia to fulminant HUS in conjunction with deep unconsciousness took less than 48 h. This close temporal relationship suggests a direct causal effect between the therapy given during surgery and anaesthesia and the recurrence of fulminant HUS. It might be hypothesised that the inflammatory response induced by surgery and anaesthesia activated cell surface receptors (e.g., CXCR4) in a patient at risk of developing HUS because of an EHEC infection, which would hasten the progression of the disease [13-16]. The noteworthy association in time between anaesthesia for uncomplicated surgery and the unique recurrence of STEC-HUS has, to our knowledge, not been described previously.

## Conclusion

Anaesthetists and surgeons should be notified of HUS/TTP as an uncommon differential diagnosis of seizures and coma in the postoperative period. Patients with a recent EHEC infection should be followed postoperatively for signs of HUS.

## Consent

A written informed consent was obtained from the patient’s next of kin for publication of this case report.

## Abbreviations

ANA: Anti-neutrophil antibody; ANCA: Anti-neutrophil cytoplasmic antibody; CT: Computer tomography; CXCR4: Chemokine receptor 4; EEG: Electroencephalogram; E.Coli: Escherichia coli; EHEC: Enterohaemorrhagic e. coli; GCS: Glasgow coma scale; HUS: Haemolytic uremic syndrome; aHUS: Atypical HUS; STEC-HUS: Shiga-toxin-producing e. coli associated HUS; MRI: Magnetic resonance imaging; PCR: Polymerase chain reaction; TMA: Thrombotic microangiopathy; TTP: Thrombotic thrombocytopenic purpura.

## Competing interests

The authors declare that they have no competing interests.

## Authors’ contributions

AM was involved in the case, reviewed the case and wrote the manuscript. MC reviewed the case and wrote the manuscript. Both authors approved the final manuscript.
